# Coronary Artery Bypass Grafting: 30-Day Operative Morbidity Analysis in 1046 Patients

**DOI:** 10.4021/jocmr1020w

**Published:** 2012-07-20

**Authors:** Nizar R. AlWaqfi, Yousef S. Khader, Khaled S. Ibrahim, Fahmi M. Eqab

**Affiliations:** a Princess Muna Heart Center, Department of General Surgery, Jordan University of Science and Technology and King Abdullah University Hospital, Irbid, Jordan; b Department of Public Health, Community Medicine, and Family Medicine, Jordan University of Science and Technology, Irbid, Jordan

**Keywords:** Bypasses, Coronary artery, Morbidity, Factor, Risk analysis

## Abstract

**Background:**

To determine the rate and risk factors of three operative complications (renal failure, pneumonia, and sternal wound infection) within 30 days after isolated coronary artery bypass surgery.

**Methods:**

Medical records of 1,046 consecutive patients between the years 2005 and 2009 were reviewed. Demographic data and peri-operative information were collected and analyzed. Univariate and multivariate analysis between different variables were performed.

**Results:**

Of all patients 3.6% developed pneumonia, 5.9% developed acute renal failure and 8.5% developed sternal wound infection. Independent predictors of acute renal failure were age > 65 years (P = 0.030), pre-operative renal impairment (P < 0.005), peripheral vascular disease (P = 0.005), emergency surgery (P = 0.043), blood transfusion (P = 0.002) mechanical ventilation > 12 hours (P < 0.005) and prolonged inotropic support (P = 0.035). Pneumonia independent predictors were female gender (P < 0.005), diabetes mellitus (P = 0.024), peripheral vascular disease (P = 0.012), emergency surgery (P = 0.007), blood transfusion (P = 0.001), mechanical ventilation > 12 hours (P = 0.005) and prolonged inotropic support (P < 0.005). Sternal wound infection independent predictors were diabetes mellitus (P = 0.017), intra- and post-operative blood transfusion (P < 0.005), and prolonged inotropic support (P = 0.006).

**Conclusion:**

Age, female gender, history of diabetes mellitus, chronic obstructive pulmonary disease, peripheral vascular disease, renal impairment, emergency surgery, per-operative blood transfusion, mechanical ventilation > 12 hours and prolonged inotropic support are associated with the 30-day complication after on-pump isolated coronary artery bypass grafting surgery.

## Introduction

Coronary artery disease (CAD) is the leading cause of cardiovascular mortality worldwide, with more than 4.5 million deaths occurring in the developing world [1]. The ability of coronary artery bypass grafting (CABG) surgery to improve overall health-related quality of life and survival has been demonstrated [2, 3]. Although operative mortality is obviously the most deleterious clinical endpoint, the evaluation of patient outcomes has become increasingly accepted as a first step to assess and improve quality of patient care [3-6]. The most significant examples of outcomes related to cardiac surgery, particularly to CABG, were reported in the United States, Canada, and Western Europe [5-9]. Despite the decrease in short-term mortality, the 30-day complication rate after CABG surgery remains high [5, 9].

The society of thoracic surgeons (STS) reported five major morbidities (stroke, renal failure, prolonged ventilation, deep sternal wound infection, and reoperation) after CABG surgery with a composite morbidity rate of 13.4% [5]. The identification of the different risk factors that affect CABG outcomes may help to decrease the occurrence of these costly complications.

In Jordan, there is no surveillance system intended to assess the outcomes of hospital care and to the best of our knowledge there is no available literature describing the profile and outcome of CABG patients. In this study, we aimed to determine the rates of three complications of isolated CABG surgery (renal impairment, pneumonia, and sternal wound infection) and to determine their associated factors among Jordanian patients treated in a teaching hospital.

## Materials and Methods

In this study, data related to all isolated CABG surgeries performed at King Abdullah University Hospital (KAUH) over five years were analyzed. It included 1,046 consecutive patients who underwent first time, on-pump, isolated CABG surgery between January, 2005 and July, 2009. Data were collected retrospectively. An assessment form was developed by the research team to abstract the necessary data from medical records. It included information on the preoperative risk factors, intraoperative variables and postoperative outcomes. This study was approved by the ethical committee of the institution where it was conducted.

### Preoperative data

Demographic data including age and sex, smoking history and health status prior to surgery were collected for all patients. Recent myocardial infarction was defined as elevation of cardiac enzymes or as evidenced by electrocardiogram (ECG) within 4 weeks before surgery. Heart failure was considered if patients were symptomatic or on anti-failure treatment. Diabetes status (taking either oral hypoglycemic agents or insulin), hypertension status (on treatment), renal dysfunction (creatinine serum level ≥ 2.0 mg/dL or on chronic dialysis), chronic obstructive pulmonary disease (COPD) (if diagnosed at any time before surgery), peripheral vascular disease (a positive history of intermittent claudication or had a documented clinical evidence of ischemia) were all recorded.

Ejection fraction (EF) was obtained from left ventricular angiogram report or the transthoracic echocardiography measurement. It was classified into three groups according to the degree of dysfunction; normal (EF ≥ 50%), mild to moderate impairment (EF between 36 and 49%) or severe impairment (EF ≤ 35%). The number of diseased coronary arteries was obtained based on the coronary angiogram report. Surgery was considered emergency if the patient was sent to the operating room within 24 hours from time of cardiac catheterization, either due to unstable angina, haemodynamic instability, or untoward event in the catheterization suite.

### Intraoperative

Information on left internal thoracic artery (LITA) harvesting was collected. It is our routine to take down a pedicled LITA for all patients whenever appropriate. Cardiopulmonary bypass (CPB) time (≥ 120 or < 120 minutes) as well as aortic cross clamp time (≥ 90 or < 90 minutes) were recorded. Intra- and post-operative blood transfusion (one unit or more) was recorded.

### Outcome variables

Duration of mechanical ventilation (≤ 12 or > 12 hours) and duration of inotropic drugs use (≤ 12 or > 12 hours) in the intensive care unit (ICU), using either one or a combination of the following drugs; dopamine, dobutamine, noradrenalin and adrenaline, were collected. These inotropic drugs were used depending on a constellation of pre- and intra-operative factors and most importantly the clinician judgment of their need. The dose was titrated according to the clinical response of each patient. Information on the following outcomes was collected: Pneumonia (diagnosed by the pulmonogist based on clinical picture and radiological studies (chest X-Ray or computerized tomography (CT) scan), renal impairment (serum creatinine level ≥ 2 mg/dL or patient required dialysis), and sternal wound infection both superficial (SSWI) and deep (DSWI). The diagnosis of SSWI and DSWI was made based on the guidelines by Center of Disease Control and Prevention (CDC) [10].

### Statistical analysis

Data were entered into computer using the Statistical Package for Social Sciences software, SPSS (SPSS Inc., Chicago, IL, USA) version 15. Participants' characteristics were described using means, standard deviations, and percentages wherever appropriate. The differences between percentages were analyzed using χ^2^ test. Multivariable logistic regression models were fitted to determine the factors associated with 30-day morbidity among patients who underwent CABG. Adjusted OR and their 95% CI were reported. A p-value of less than 0.05 was considered statistically significant.

## Results

### Participants’ characteristics

This study included 1046 patients (252 females and 794 males). The age of patients ranged between 19 and 85 years with a mean ± standard deviation (SD) of 59.7 ± 10.1. Less than one third (30.4%) aged 55 year or less, 40.7% aged between 56 and 65, and 28.9% aged more than 65 years. More than half (54.2%) were smokers. Preoperatively, 15.2% had recent myocardial infarction, 15.8% had COPD, 65.9% had hypertension, 52.8% had diabetes mellitus, 22.8% had heart failure, 18.2% had peripheral vascular disease, and 4.6% had renal impairment. EF was > 50% in 59.3% of patients, between 36 and 49% in 26.6% of patients, and ≤ 35% in 14.1% of patients. More than two thirds (74.0%) had had three-vessel disease, 21.9% had two-vessel disease, and 2.1% had one-vessel disease. LITA was used in 98.9% of patients. Emergency surgery was performed in 11.8% of patients. About 6.7% needed prolonged inotropic support and 21.0% were on the ventilator for more than 12 hours.

### Surgery outcomes

Of all patients who underwent surgery, 3.6% developed pneumonia, 5.9% developed acute renal failure, and 8.5% developed sternal wound infection, of those about 10% had DSWI. About 17.5% stayed in the hospital for more than 10 days and 20.7% stayed in the ICU for more than 2 days. The 30-day operative mortality rate was 5.9%.

### Univariate analysis

The distribution of the surgery outcome variables according to demographic, clinical, and surgery characteristics are shown in Table 1. Patients who had recent myocardial infarction, COPD, and preoperative renal impairment were more likely to develop acute renal failure. Patients with diabetes were more likely to develop pneumonia and sternal wound infection. Pedicled LITA harvesting was not associated with increased risk of wound infection (P = 0.690). The rate of pneumonia was higher among females compared to males (7.9% versus 2.3%). Patients who underwent emergency surgery were more likely to develop renal failure and pneumonia. The rates of the three studied complications were higher among those who needed blood transfusion and those who had prolonged inotropic support in the ICU. Mechanical ventilation ≥ 12 hours was associated with higher rates of renal failure and pneumonia.

**Table 1:** The Rates of the 30-Day Complications After Coronary Artery Bypass Grafting in 1046 Jordanian Patients According to Demographic, Clinical, and Surgery Characteristics

Patients characteristics	Renal failure n (%)	Pneumonia n (%)	Sternal wound infectionn (%)
Age (year)			
≤ 55	12 (3.8)	12 (3.8)	24 (7.5)
56 - 65	26 (5.1)	14 (3.3)	38 (9.0)
> 65	24 (7.9)	12 (4.0)	27 (8.9)
Sex			
Female	20 (7.9)	20 (7.9)*	27 (10.7)
Male	42 (5.3)	18 (2.3)	62 (7.8)
Myocardial infarction			
Yes	19 (11.9)*	4 (2.5)	9 (5.7)
No	43 (4.8)	34 (3.8)	80 (9.0)
Peripheral vascular disease			
Yes	25 (13.2)*	8 (4.2)	12 (6.3)
No	37 (4.3)	30 (3.5)	77 (9.0)
Smoking			
Yes	35 (6.2)	17 (3.0)	37 (6.5)
No	27 (5.6)	21 (4.4)	52 (10.9)
Chronic obstructive pulmonary disease			
Yes	17 (10.3)*	10 (6.1)	12 (7.3)
No	45 (5.1)	28 (3.2)	77 (8.8)
Hypertension			
Yes	45 (6.5)	30 (4.4)	57 (8.3)
No	17 (4.8)	8 (2.2)	23 (9.0)
Diabetes			
Yes	36 (6.5)	29 (5.3)*	61 (11.1)*
No	26 (5.3)	9 (1.8)	28 (5.7)
Renal impairment			
Yes	14 (29.2)*	0 (0)	2 (4.2)
No	48 (4.8)	38 (3.8)	87 (8.7)
Ejection fraction			
≥ 50	36 (5.8)	22 (3.5)	47 (7.6)
36 - 49	14 (5.0)	12 (4.3)	31 (11.2)
≤ 35	18 (12.2)	18 (12.2)	11 (7.4)
Emergency			
Yes	18 (14.6)*	11 (8.9)*	14 (11.4)
No	44 (4.8)	27 (2.9)	75 (8.1)
Pump time (minutes)			
≥ 120	6 (3.8)	5 (3.2)	8 (5.1)
< 120	56 (6.3)	33 (3.7)	81 (9.1)
Aorta clamp (minutes)			
≥ 90	2 (3.3)	0 (0)	0 (0.0)
< 90	60 (6.1)	38(3.9)	89 (9.1)
Blood transfusion			
Yes	54 (8.9)*	36 (5.9)*	67 (11.0)*
No	8 (1.8)	2 (0.5)	22 (5.1)
Inotropic support			
Yes	14 (20)*	16 (22.9)*	13 (18.6)*
No	48 (4.9)	22 (2.3)	76 (7.8)
Ventilation > 12 hours			
Yes	34 (15.5)*	24 (10.9)*	21 (9.5)
No	28 (3.4)	14 (1.7)	68 (8.3)
Number of grafted coronaries			
1	1 (4.5)	1 (4.5)	3 (7.5)
2	14 (6.1)	6 (2.6)	20 (8.7)
3	46 (5.8)	46 (4.9)	66 (8.5)

*Significant (P < 0.05)

### Multivariate analysis

The predictors of 30-day operative complications are shown in Table 2. Factors that were significantly associated with renal failure were age, peripheral vascular disease, renal impairment, emergency CABG, intra- and post-operative blood transfusion, ventilation ≥ 12 hours, and prolonged inotropic support. Patients aged > 65 years were significantly more likely to develop renal failure compared to patients aged ≤ 55 years (OR = 2.2; 95% CI: 1.1, 4.5). Patients with pre-operative renal impairment were six times (OR = 6.1; 95% CI: 2.9, 13.1) and patients with peripheral vascular disease were almost twice (OR = 2.3; 95% CI: 1.3, 4.2) more likely to develop renal failure. Emergency CABG, blood transfusion, ventilation ≥ 12 hours, and prolonged inotropic support were significantly associated with increased odds of renal failure.

**Table 2:** The Multivariate Analysis of Factors Associated With the 30-Day Complications After Coronary Artery Bypass Grafting in 1046 Jordanian Patients

Variable	Renal failure		Pneumonia		Sternal wound infection	
	OR (95% CI)	P-value	OR (95% CI)	P-value	OR (95% CI)	P-value
Age (year)						
≤ 55	1.0					
56 - 65	1.7 (0.8, 3.3)	0.157				
> 65	2.2 (1.1, 4.5)	0.030				
Gender (female vs. male)			4.1 (2.0, 8.3)	< 0.005		
Diabetes mellitus			2.4 (1.1, 5.3)	0.024	1.8 (1.1, 2.9)	0.014
Chronic obstructive pulmonary disease			2.8 (1.3, 6.4)	0.012		
Peripheral vascular disease	2.3 (1.3, 4.2)	0.005				
Renal impairment	6.1 (2.9, 13.1)	< 0.005				
Emergency surgery	2.0 (1.0, 3.8)	0.043	2.8 (1.3, 6.0)	0.007		
Blood transfusion	3.5 (1.6, 7.9)	0.002	11.3 (2.6, 48.2)	0.001	2.1 (1.3, 3.4)	0.004
Ventilation ≥ 12 hours	3.2 (1.8, 5.8)	< 0.005	3.0 (1.4, 6.4)	0.005		
Prolonged inotropic support	2.3 (1.1, 4.9)	0.035	7.3 (3.3, 16.1)	< 0.005	2.3 (1.2, 4.4)	0.013

Pneumonia was significantly associated with gender, diabetes mellitus, COPD, emergency CABG, blood transfusion, ventilation ≥ 12 hours, and prolonged inotropic support. Females were almost four times more likely to develop pneumonia compared to males (OR = 4.1; 95% CI: 2.0, 8.3). Having diabetes mellitus (OR =2.4; 95% CI: 1.1, 5.3) and peripheral vascular disease (OR =2.8; 95% CI: 1.3, 6.4) were significantly associated with increased odds of pneumonia. Emergency CABG, blood transfusion, ventilation ≥ 12 hours, and prolonged inotropic support were significantly associated with increased odds of pneumonia.

Sternal wound infection was significantly associated with diabetes, blood transfusion, and prolonged inotropic support. Diabetes was associated with increased odds of sternal wound infection by 80%. Blood transfusion and prolonged inotropic support were significantly associated with increased odds of sternal wound infection.

## Discussion

Open heart surgery has been performed at our center for less than a decade, with an annual increase in number and diversity of performed procedures. This is the first report describing the 30-day operative complications following isolated coronary surgery and their predictors. Pneumonia, renal impairment, and sternal wound infection within 30 days after surgery were the studied outcomes. Patients who died with any of these end points were also included. Of all patients, 3.6% developed pneumonia, 5.9% developed renal failure, and 8.5% developed sternal wound infection. These findings were fairly similar to earlier studies [9, 11, 12].

Prolonged inotropic support in the ICU and per-operative transfusion of one or more packed red blood cells units (PRBC), were predictors of the three end points. Both of these factors are reflections of low cardiac output syndrome with resultant tissue hypoperfusion. Besides, blood transfusion significantly increases the risk of respiratory failure and ICU stay [13]. Measures directed to reduce blood loss during surgery may help reduce the need for blood transfusion: reduction of CPB prime volume, meticulous surgical haemostasis, and utilization of autologous blood transfusion.

Concern for high operative complications among old age group was raised in different previous reports [14, 15]. In our multivariate analysis, patients > 65 years were found to be at an increased risk of developing renal failure, but not pneumonia or surgical wound infection. Stoica SC et al [15] reported a significantly increased risk of developing post-operative renal failure in the elderly. This group of patients may be susceptible to many forms of acute renal failure because an aging kidney loses function and the ability to withstand acute insults and may have reduced ability to cope with critical circulation [16].

In the major morbidity risk models developed, the impact of female gender was not consistent across different complications. For example; the impact of female gender appears to be slightly protective for the major morbidity endpoints of renal failure requiring dialysis [17]. However, female gender appears to predispose to higher adverse event rates for the outcomes of prolonged ventilation, and deep sternal wound infection [5]. Female gender in our study was an independent predictor of developing post-operative pneumonia. However, the incidence of post-operative renal impairment and sternal wound infection was not affected by gender difference.

Many studies have suggested the importance of chronic comorbidities (COPD, pre-operative myocardial infarction, hypertension, diabetes mellitus, peripheral vascular disease, and renal impairment) in predicting the outcome of cardiac operations. Conflicting results have been reported in different studies regarding the impact of these different comorbid conditions and their reflection on the operative complications [5, 8, 9, 11-13]. This may be related to the lack of sufficiently large numbers that may minimize the potential effects of chance or may be due to the different prevalence of comorbidities among different populations. In our series, history of smoking and some of the studied pre-operative comorbidities were not significant predictors of post-operative complications. The smaller sample size of some of the above mentioned comorbidities have contributed to the non-predictive strength of these risk factors.

Diabetes has long been described as an independent risk factor for the development of coronary artery disease, and the proportion of diabetic patients undergoing CABG is steadily increasing [8]. As with other reports [18-22], diabetic patients were found to have significantly higher incidences of post-operative complications. Diabetics had higher risk of developing post-operative pneumonia and sternal wound infection, but not acute renal failure. Chukwuemeka et al [21] found no correlation between diabetes mellitus and the development of post-operative renal failure. Previous studies have reported an association between diabetes and sternal wound infections [21, 22].

Patients with COPD and those who spent > 12 hours on mechanical ventilation were at a significantly increased risk of developing post-operative pneumonia. Also, prolonged mechanical ventilation was an independent predictor of post-operative renal failure, confirming previous reports [23-25]. This group of patients has an impaired physiologic reserve and function of their lungs, in addition to the burden of being at an increased risk of sepsis, due to the prolonged ventilation and ICU stay. Filsoufi et al [25] reported a three-times-increased rate of respiratory failure in patients with a history of COPD and a significantly increased risk of renal failure requiring dialysis. Although this is a single center study, the association with the duration of ventilation should be interpreted carefully, because the duration of ventilation is often affected by other factors unrelated to the patient. Not adjusting for these unmeasured variables is an important source of bias in epidemiologic studies.

In the present study, our data showed that peripheral vascular disease was an independent predictor of post-CABG renal failure. Stallwood et al [18] reported the role of peripheral arteriopathy on post-CABG renal failure. This may be the result of renal parenchymal disease or renal artery stenosis [26]. On the other hand Chu et al [27] concluded that patients with peripheral vascular disease had similar incidence of post-CABG renal failure when compared to patients without peripheral vascular disease.

A higher than normal serum creatinine level was associated with an increased risk of both, post-operative renal failure and sternal wound infection by univariate analysis, and it was an in dependent predictor of post-operative renal failure requiring dialysis by multivariate analysis. This is consistent with previous peer reviews [18].

Ahmadi et al [28] reported an increased risk of post-operative renal failure in patients taken for CABG surgery on emergency basis. Our emergency cases were at a significantly increased risk to develop post-operative pneumonia and renal failure but not sternal wound infection. The majority of these patients had a preoperative cardiac status instability that may compromise renal perfusion and enhance the effect of renal ischemic events that occur during CPB.

In conclusion, age, female gender, a history of diabetes mellitus, COPD, peripheral vascular disease, renal impairment, emergency surgery, per-operative blood transfusion, mechanical ventilation > 12 hours and prolonged inotropic support are associated with the 30-day complications after on-pump isolated CABG surgery.
